# Toxins in Fermented Foods: Prevalence and Preventions—A Mini Review

**DOI:** 10.3390/toxins11010004

**Published:** 2018-12-24

**Authors:** Bhagavathi Sundaram Sivamaruthi, Periyanaina Kesika, Chaiyavat Chaiyasut

**Affiliations:** Innovation Center for Holistic Health, Nutraceuticals, and Cosmeceuticals, Faculty of Pharmacy, Chiang Mai University, Chiang Mai 50200, Thailand; p.kesika@gmail.com

**Keywords:** toxins, fermented foods, mycotoxins, bacterial toxins, lactic acid bacteria, fermentation

## Abstract

Fermented foods (FF) are widely consumed around the world, and FF are one of the prime sources of toxins and pathogenic microbes that are associated with several foodborne outbreaks. Mycotoxins (aflatoxins, fumonisins, sterigmatocystin, nivalenol, deoxynivalenol, zearalenone, ochratoxin, and alternariol), bacterial toxins (shiga toxin and botulinum), biogenic amines, and cyanogenic glycosides are the common toxins found in FF in addition to the pathogenic microbes. Fermented milk products and meat sausages are extremely vulnerable to contamination. Cumulative updated information about a specific topic such as toxins in FF is essential for the improvement of safer preparation and consumption of fermented foods. Accordingly, the current manuscript summarizes the reported mycotoxins, bacterial toxins, and/or toxins from other sources; detection methods and prevention of toxins in FF (use of specific starter culture, optimized fermentation process, and pre- and post-processing treatments); and major clinical outbreaks. This literature survey was made in Scopus, Web of Science, NCBI-PubMed, and Google Scholar using the search terms “Toxins” and “Fermented Foods” as keywords. The appropriate scientific documents were screened for relevant information and they were selected without any chronological restrictions.

## 1. Introduction

Fermented foods (FFs) are an important part of the Asian diet. It has been predicted that the global market for fermented products and ingredients may reach up to $28.4 billion by 2022. The growing world population increases the need for food, and increased awareness and evidence of the beneficial effects of FF have increased the demand for fermented products [1]. FF are rich in bioactive microbes, and the concentration and diversity of the microbial load in the FF are different. Microbes isolated from FF have been reported for several biological properties like bacteriocin, enzyme and neurotransmitter production, and probiotic activities [2]. The production process of FF also varies based on the geographical region and availability of the resources. In the ancient period, almost all the FF were prepared by spontaneous or blind (not aware of the participating microbes) fermentation processes. The specific starter cultures were used to control the fermentation process, and lactic acid bacteria (LAB) was commonly used as a starter culture. Some of the food materials such as milk naturally harbor some LAB strains, which also play a role in the fermentation process and are considered as non-starters [3].

The production procedure varies based on the nature of the product. The traditional preparation of torba yogurt has the following steps: 1. Collectoin of milk and subjecting milk to heat to pasteurize it. 2. The pasteurized milk is cooled to the desired temperature. 3. Inoculation of previous yogurt (acts as a seed culture). 4. The mixture is incubated. 5. The fermented milk is cooled to room temperature. 6. After mixing gently, the leftovers (whey) is strained, and the torba yogurt is stored. Ayran, a yogurt-based beverage, is also made from milk using specific starter cultures (*Streptococcus thermophilus*, *Lactobacillus delbrueckii* subsp. bulgaricus). Likewise, every fermented product is prepared with different processing methods, even when they have a common base ingredient. For example, milk is the base ingredient of Koumiss, Kefir, Ayran, Torba, and Kurut, which are prepared with different processing methods [4]. The commonly used, traditional, and patented approaches of fermented food preparations have been detailed previously [5,6,7].

The fermentation process provides several advantages to the food products such as flavor, taste, shelf life, etc. The fermenting microbes can release their bioactive metabolites such as vitamins, peptides, exopolysaccharides, conjugated linoleic acids, neurotransmitters (γ-aminobutyric acid; GABA), and oligosaccharides [8]. On the other hand, some unwanted toxic contaminants and metabolites of the microbes are released into the food base during fermentation. Mycotoxins, bacterial toxins, biogenic amines, and cyanogenic glycosides are the frequently occurring toxic compounds in FF. 

The presence of excess amounts of undesirable toxic compounds in the FF beyond the approved level could lead to serious health issues among the consumers or even cause mortality. The occurrence of pathogenic bacteria (*Brucella* spp., *Listeria* spp., *Salmonella* spp., *Staphylococcus aureus*, *Streptococcus* spp., and *Clostridium botulinum*) in FF, especially in fermented milk products, have caused serious outbreaks [9]. The quality of the FF becomes questionable, and that affects the entire FF market, if the toxic incidences occur often. Thus, several precautions were made in the food processing industries to ensure high-quality product making and safe maximum residual levels of pathogenic microbes and toxins in the food products. 

This current review summarizes the common toxins in FF and their sources and prevention measures along with a short note on clinical outbreaks of FF. The literature survey was made in Scopus, Web of Science, NCBI-PubMed, and Google Scholar using “Toxins” and “Fermented Foods” as keywords. The appropriate scientific documents (research article, review papers, short communications, and opinions) were screened for the relevant information and they were selected without any chronological restrictions.

## 2. Common Toxins in Fermented Foods

### 2.1. Mycotoxins

All fungal toxins are collectively called mycotoxins. Several mycotoxins (aflatoxins, ochratoxin A, zearalenone, deoxynivalenol, and citrinin) were reported to be present in FF. The fungal toxins in South African traditionally brewed beers were investigated. The grains, barley, and maize used for the beer production, locally brewed and commercial alcohol beverages, were screened for the mycotoxin producer, and it was discovered that the grains contain *Aspergillus* spp., *Penicillium* spp., *Mucor* spp., and *Rhizopus* spp., and some commercial beverages contained aflatoxins while some local beverages contained ochratoxin A and zearalenone [10].

Attieke, a traditional Ivorian fermented food, can be produced using mold-covered cassava inoculum. The inoculum and attieke samples were subjected to screening of mycotoxins. The results suggested that ochratoxin (0.2 µg/kg) and deoxynivalenol were present in the sample in trace amounts, and the biological testing study revealed that the consumption of attieke is safe [11]. The Nigerian food samples (maize, millet, Sorghum, sesame, fermented cassava, and flakes) were screened for ochratoxin A contamination. About 98% of the samples contained ochratoxin A, and more than 74% of samples contained > 5 µg of toxin/kg of the sample, which is regarded as unsafe to human health as per the European Union standard [12]. Ochratoxin A is a potent carcinogen [13] and nephrotoxin [14]. The presence of mycotoxins in wine, cider samples, and cork stoppers was analyzed by liquid chromatography-tandem mass spectrometry. The type and concentration of the toxins varied among samples. Some wine samples contained ochratoxin A while some of them contained alternariol and alternariol methyl. The cork stoppers of red wine samples were reported with the presence of penicillic acid, alternariol, and alternariol methyl. The results suggested that the packing process of fermented foods needed more critical care [15]. Alternariol is a mutagen [16] and is genotoxic [17], cytotoxic, teratogenic, and fetotoxic [18]. 

*Rhizopus* spp. are commonly used to produce Asian fermented foods. Some of the *Rhizopus* spp. have become opportunistic pathogens for immunocompromised consumers, some other *Rhizopus* spp. have an endosymbiotic association with *Burkholderia*, which can produce toxic metabolites. Screening of several *Rhizopus* strains revealed that about 11% of strains had an endosymbiotic relationship with *Burkholderia* spp. Those strains were proven to have the ability to produce rhizoxins. Therefore, the careful screening and identification of pathogenic and toxigenic associations with commonly used mold in the food industry is obligatory [19]. Rhizoxins are potent antitumor agents [20,21].

The traditional Nigerian condiments *iru* and *ogiri* and their raw materials, locust bean and melon seeds, respectively, were studied for mycotoxins and microbial contaminations. Pathogens such as *Bacillus anthracis*, *Staphylococcus sciuri* subsp. sciuri, *Alcaligenes faecalis*, and *Proteus mirabilis* were found in many of the samples, and about 25% and 23.5% of pathogens detected in the samples belonged to *Bacillus*, and *Staphylococcus* spp. family, respectively. The results suggest that some of the Nigerian raw materials and condiments are severely contaminated with deadly pathogens. Aflatoxin was found in *ogiri* and melon while there were no toxins in other samples. The level of mycotoxin was low in the studied food samples [22]. In another study, Southwest Nigerian FF samples (maize gruel, sorghum gruel, locust bean, melon seeds, and African oil bean seeds) were found to contain mycotoxins. About 82% of tested samples had mycotoxin contamination. Fumonisin B1 was found to be dominantly present in sorghum gruel samples, where African oil bean seeds had aflatoxin B1 (3–36 µg/kg), and sterigmatocystin. One of the samples was found with multi-mycotoxin contamination. The studies claimed that people were not aware of toxins, their consequences, and the unhygienic practices [23]. Aflatoxin is a potent carcinogen and is an immunotoxic and hepatotoxic compound [24,25].

Meju, a fermented soybean sample, was screened for fungal contamination. Meju prepared with ethanolic extracts of *Nelumbo nucifera*, *Allium sativum*, and *Ginkgo biloba* was studied. The results showed that Meju samples contained about ten fungal species, which came under the genera *Agaricaceae, Mucor, Penicillium, Aspergillus*, and *Paecilomyces*. Meju prepared without plant extracts contained aflatoxin B1 and the ochratoxin A producing fungus, *Aspergillus ruber* [26].

Fermented milk products are predominantly contaminated with aflatoxins. The fermentation and storage temperature, storage time, acidity, pH, heat processes, aflatoxin concentration, and strain used for the fermentation process are the major factors that influence the free aflatoxins in fermented milk products [27] (Table 1). The prevention of the occurrence of free aflatoxins in fermented milk products is detailed in the later part of this review. 

### 2.2. Bacterial Toxins

*Bacillus* spp. isolated from *Soumbala* (fermented *Parkia biglobosa* seeds) and *Bikalga* (fermented *Hibiscus sabdariffa* seeds) have shown enterotoxin production. *B. cereus* isolates are hemolysis positive and produce enterotoxins in both laboratory media and in fermented products [28]. Recently, Agbobatinkpo et al. [29] reported the prevalence of anerobic-spore forming *Bacillus* spp. in fermented *Hibiscus sabdariffa* seeds. They reported that among 355 isolates, *B. subtilis, B. cereus, B. amyloliquefaciens, B. licheniformis, B. safensis, B. altitudinis, B. aryabhattai, B. flexus*, and *B. circulans* were predominant species. 

Yellow-water is a food flavor-enhancer, a byproduct of Chinese traditional brewing liquor. Among the 50 samples, 17 samples contained toxigenic *B. cereus* contamination. More than 90% of the isolates were positive for atleast two genes of the toxin coding genes. The study suggested that the microbiological quality of yellow-water needs improvement [30]. 

*Gergoush* (a fermented Sudanese bread snack) is made by several fermentation processes using different starter cultures. A series of screenings on *Bacillus* spp. predominance in the starter cultures used to produce *gergoush* revealed that *B. cereus sensu lato* (40–68%), *B. licheniformis* (16–27%), *B. subtilis* (8–32%), and *B. sonorensis* (4–20%) are the common contaminants. However, no viable cells were found after the baking process, so the study proved that the *gergoush* was microbiologically safe [31]. About eighty-seven *B. cereus sensu lato* strains were isolated from representative Korean fermented soybean products (*Doenjang, ssamjang, kochujang*, and *cho-kochujang*). The strains were positive for the enterotoxin coding genes in PCR analysis and were resistant to most of the β-lactam antibiotics and could potentially cause diarrheal diseases [32]. The commercial Korean fermented soybean paste (*doenjang*) samples were studied for the presence of enterotoxigenic *B. cereus* strains. About 35 isolates were isolated from 67 samples, among which 12 isolates were positive for the emetic toxin and were highly resistant to antibiotics. The study suggested that the rep-PCR based screening of *Bacillus* strains may help to differentiate the emetic and non-emetic strains, and that extra care is needed while preparing the fermented soybean product [33]. Park et al. [34] also reported the occurrence of toxigenic *B. cereus* strains in *doenjang* [34]. The emetic toxin causes vomiting and nausea upon ingestion of the contaminated food [41,42] and also causes liver failure [43].

The commercial fresh and fermented dairy products (yoghurt, *doogh*, and *kashk*) of Iran have been screened for shiga toxin-producing *Escherichia coli* (STEC) contamination. About 8% of the samples were positive for STEC, and most of them came under the subgroups of O157 and O26. The isolates displayed multi-drug resistance against tested antibiotics. The study suggested that dairy products are easily contaminated with drug resistant STEC strains [35]. Likewise, fresh and fermented dairy products of Ogun State, Nigeria have tested positive for the occurrence of the STEC O157 strain. Among the 202 screened samples, 10 samples contained STEC O157. The isolates were also resistant to several common antibiotics [36]. 

The milk products of East and West African regions shelter pathogenic human and livestock-associated *Staphylococcus aureus* strains (HLSA). A detailed study proved that the HLSA strains are encoded with Panton-Valentine leukocidin, toxic shock syndrome toxin, and enterotoxins. The study suggested that hygienic practices in food processing and preservation are the only way to prevent infection [37].

The traditional North Central Nigerian FF samples (*ogi, nono, wara, kunu, iru*, and *kindirmo*) were screened for the presence of coagulase-negative staphylococci strains (CNS). About 255 CNS strains were isolated and characterized. Most of the isolates belonged to *Staphylococcus epidermidis*, *S. simulans, S. xylosus, S. kloosii*, and *S. caprae*. The virulence screening study suggested that some of the CNS isolates can produce enterotoxin and can cause serious health issues to immune compromised people [38].

*B. cereus* that is isolated from *iru* and *ogiri* has the potential to produce diarrheal toxins [39]. Adedeji et al. [22] screened the Nigerian traditional condiments (*iru* and *ogiri*) for toxic compounds and microbial contamination. The result showed that the samples of *iru* and *ogiri* were deeply contaminated with enterotoxigenic microbes such as *Alcaligenes faecalis*, *Proteus mirabilis*, *Staphylococcus sciuri* subsp. *sciuri*, and *Bacillus anthracis*. The study claimed that the people who prepare the FF in the south-western Nigerian region are unaware of hygienic practices [22].

*Clostridium botulinum* is the causative agent of botulism, a paralytic disease caused by bacterial neurotoxins. Several foodborne botulism cases have been reported [44,45]. Fermented fish products are a shelter for *C. botulinum* in many cases. *Muktuk* (whale meat product) consumption caused botulism outbreaks in Alaska and the Canadian Arctic regions. Likewise, the consumption of *rakfisk*, *faseikh*, *kapchunka*, tofu, and *ashbal* have been associated with outbreaks of botulism in Egypt, Israel, New York, China, and Japan [40] (Table 1).

### 2.3. Other Toxic Materials in FF

Biogenic amines (BA) are another major toxic entity in FF. Fish, fish products, and FF have been detected with a high amount of BA, and this causes serious health issues to consumers. The presence and level of BA in food material is an indicator of microbial activity (microbial decarboxylation) in stored or processed foods. Histamine, putrescine, tyramine, cadaverine, and β-phenylethylamine are the common BA in FF, specifically, histamine is accountable for several food poisonings and outbreaks. Unhygienic manufacturing and storage practices are the reason for histamine poisoning in fish products. Several *Lactobacillus* spp., *Bacillus* spp., *Bacillus subtilis* strains, *Staphylococcus* spp., *Streptococcus* spp., and *Enterococci* spp. can produce BA in FF such as sausages, wine, *natto*, *miso*, and *douchi* [46]. The source, factors affecting the formation, toxicological properties, detection, and prevention of BA, and BA content in foods have been reported previously [46,47,48,49]. 

Some of the plant toxins such as cyanogenic glycosides have been reported in FF (fermented bamboo shoots). The traditional methods of cooking effectively reduced the cyanide toxins from several bamboo shoot-based foods (*soidon, soibum*, and *soijin*) [50].

## 3. Detection Methods

Several conventional detection methods are available for detecting the presence of pathogens and toxins in FF. Culture-dependent methods (growing the microbes on laboratory media and characterization), biochemical profiles, microscopic observations, and PCR were commonly used for microbial detection. Immunology-based methods such as ELISA, Limulus amoebocyte lysate, rabbit pyrogen test, and chromatography techniques were employed for the detection of toxins in FF. Recently, many biosensors have been in use to detect the contamination in food processing and storage. Different bioreceptors (such as enzymes, antigen/antibody, phages, and nucleic acids) were used in the biosensors to improve the efficiency and accuracy. Optical, electrochemical, nanomaterial-based sensors exist to detect the toxic substances in foods. A detailed review of biosensors used for the detection of pathogens and toxins has been reported recently [51]. 

A PCR-based detection of endosymbionts of *Rhizopus microsporus*, which is commonly used to produce *tempe* and *sufu*, has been reported. The metabolic profile of the symbiotic fungal-bacterial complex indicated that endosymbiontic bacteria produces antimitotic agents. A quick PCR validation (ketosynthase gene) of the starter cultures used to produce soybean fermented product can be a safe way to detect the toxicogenic symbiotic bacteria [52]. Møller et al. [53] proved that NMR (Nuclear Magnetic Resonance) relaxometry is a reliable and propitious technique for monitoring the microbial safety in fermented meat production.

Shiga toxin producing *E. coli* (STEC) strains can be detected by PCR. The primers of *rpoB*, *eaeA*, *stx1*, and *stx2* genes were used for the promising detection of STEC in meat and dairy samples. The study on 54 STEC samples proved that most of the fermented milk and meat products were contaminated with STEC [54]. Similarly, the multiplex PCR method has been proposed for the detection of Verocytotoxin-producing *Escherichia coli* (VEC) O26 (VECO26) in beef and milk samples. The primers were designed for the specific amplification of *stx1* (verocytotoxin type 1), *stx2* (verocytotoxin type 2), and *wzx* (O26 antigen) genes. The results were accurate and reproducible. Moreover, no false positive results were obtained. Thus, the proposed PCR method can be used to detect the presence of VECO26 in milk and beef products [55]. A logarithmic model to predict the VEC in fermented raw-meet sausages (FRMS) was developed and evaluated during processing and storage of dry and semidry FRMS. The computing tool was named EcSF (E. coli safeFerment), and it is freely available. The developers argued that EcSF could be a reliable computational method to monitor the FRMS during processing and storage [56]. 

An immunomagnetic bead-based pretreatment and ELISA techniques have been validated to determine the presence of trace amounts of aflatoxin B1 in fermented soybean sauces. The results suggested that immunomagnetic bead treatment was better than conservative extraction and immunoaffinity validation processes. The detection range, linear relationship, sensitivity, precision, and operation methods suggest that the immunomagnetic bead-based detection of aflatoxin B1 is a reliable technique [57].

## 4. Prevention Measures

### 4.1. Use of Lactic Acid Bacteria

The use of lactic acid bacteria (LAB) to eliminate the mycotoxins in FF is a common practice in various food industries. The strains of *Lactobacillus* spp., *Bifidobacterium* spp., *Lactococcus* spp., *Leuconostoc* spp., *Pediococcus* spp., *Propionibacterium* spp., and *Streptococcus* spp. have been routinely used to remove the mycotoxins in foods, and these strains are known for their antagonistic and toxin binding ability. The aflatoxin eradicating ability of LAB strains have been reported recently [58].

An anti-aflatoxin producing *Aspergillus* spp. activity was reported for the following *Lactobacillus* strains (*Lactobacillus fermentum* OYB, *L. fermentum* RS2, *L. plantarum* MW, *L. plantarum* YO, *L. brevis* WS3, and *Lactococcus* spp. RS3.) isolated from fermented cereal gruels. *L. plantarum* YO inhibited the food-contaminating aflatoxin B1 (AB1) and G-producing *Aspergillus* spp. *in vitro*. The results suggested the use of antagonistic *Lactobacillus* spp. to reduce or prevent the mycotoxins in FF products [59].

LAB (*Streptococcus lactis* and *Lactobacillus delbrueckii*) mediated fermentation processes reduced the level of spiked fumonisin B1 and zearalenone (ZEA) in fermented maize meal (FMM). The cytotoxicity, tested in the SNO cell line, of the LAB-mediated FMM was reduced significantly when compared to FMM without LAB. The results suggested that LAB mediated fermentation reduced or removed the mycotoxins in FMM, but the mechanism of the beneficial effect is not known [60], and it is likely that the level of AB1 also reduced significantly during the preparation of LAB-mediated FMM [61].

A *Saccharomyces cerevisiae* strain that was isolated from indigenous fermented foods of Ghana exhibited high affinity binding to AB1. About 40% of added AB1 was attracted by the *S. cerevisiae* strain, and the surface binding was not affected by growth temperature. Non-viable cells of the *S. cerevisiae* strain exhibited significant AB1 affinity. The results suggested that care should be taken when selecting the starter culture during the preparation of FF [62]. 

The aflatoxin M1 (AM1) binding efficiency of *Lactobacillus casei* in kefir samples was evaluated. The use of *L. casei* in kefir preparation effectively absorbed 88.17% of the added AM1. *L. casei* synergistically works with kefir starter culture to reduce the AM1. The studied *L. casei* has the potential to detoxify the AM1 when used in kefir production [63]. 

The *B. subtilis* HJ18-4 strain isolated from *sokseongjang* (Korean fermented soybean) exhibited antagonistic activity against food contaminant *B. cereus*. The co-cultivation study proved that *B. subtilis* HJ18-4 effectively down-regulated the expression of toxin coding genes in *B. cereus*. The aqueous extract of the HJ18-4 fermented soybean product significantly suppressed the growth of *B. cereus* [64]. Likewise, another isolate of *doenjang*, *B. amyloliquefaciens* RD7-7, exhibited antagonistic activity against *B. cereus* and suppressed the expression of toxin coding genes [65]. The results indicated that the strains HJ18-4 and RD7-7 are potent candidates to prevent *Bacillus* contamination in various food industries.

*Lactobacillus plantarum* strains, isolates of several FF, were screened for ZEA-degrading ability *in vitro*. The strains *L. plantarum*4, *L. plantarum*22, and *L. plantarum*39 showed about 39, 47, and 38% of ZEA degradation, respectively. The elimination of ZEA was influenced by the concentration of toxins, temperature, and viability of the cells. Though ZEA elimination of *L. plantarum* is slow, it is a continuous process. So, the use of the studied *L. plantarum* strains helped to reduce the ZEA contamination in FF [66].

The use of *Lactobacillus sakei* KM5474 and *L. plantarum* KM1450 successfully reduced the biogenic amines (BA) in *som-fug* (Thai fermented fish sausage). Histamine, tyramine, and putrescine are frequently occurring BAs in *som-fug*. The use of one or both strains (KM5474, and KM1450) reduced the BA formation, but the addition of two strains had better BA removal activity compared to a single strain in *som-fug* [67].

LAB strains isolated from *cheonggukjang* (fermented soybean paste) were studied for their tyramine-reducing ability. The strains *B. amyloliquefaciens* D31-21, *B. amyloliquefaciens* O2-2, and *B. subtilis* H3 exhibited potent tyramine-reducing activity. The *cheonggukjang* prepared with *B. subtilis* H3 showed maximum tyramine reduction. The results suggest that *B. subtilis* H3 can be used to produce tyramine-free *cheonggukjang* [68].

The anti-AB1 activity of *Lactobacillus plantarum* C88 strain isolated from *tofu* was evaluated using a rodent model system. The strain C88 exhibited high AB1-binding ability *in vitro*. The oral administration of a single dose (4.0 × 10^10^ CFU) of C88 culture significantly improved the antioxidant system of the AB1-damaged mice and facilitated the excretion of AB1 via feces. The histopathological studies also suggested that the intervention of C88 improved the health status of toxin-damaged mice [69].

Two *Aspergillus* strains (*Aspergillus oryzae* MAO103, *A. oryzae* MAO104) isolated from *Meju* can effectively degrade AB1 by up to 90% in two weeks and also suppress the growth of AB1 producing *Aspergillus flavus*. The results suggest that *A. oryzae* MAO103 and *A. oryzae* MAO104 strains can be used in various food industries to control the AB1 level [70]. 

The use of LAB-based starter cultures significantly inhibited the growth of pathogenic strains of *E. coli* in sausages. The starter culture used, bacterial enzymes, and ripening time are influential factors that affect the release of free amino acids during sausage preparation. The efficiency of single or mixed strains of *Staphylococcus carnosus. L. sakei, L. plantarum, Pediococcus pentosaceus*, and *P. acidilactici* in sausage production was demonstrated recently [71]. *L. plantarum* LUHS135 and *L. paracasei* LUHS244 isolated from fermented cereal foods exhibited antagonistic activity against several common food-borne pathogens and also reduced the mycotoxins effectively. The strains LUHS135, and LUHS244 are pH tolerable, sensitive to antibiotics, and can ferment many carbohydrates. The encapsulation process increased the stability of the strains. Collectively, the results suggest that the strains LUHS135 and LUHS244 are more suitable, as a bio-controlling agent, for industrial-scale food preparation [72].

### 4.2. Processing Conditions 

The improvement of the fermentation process and updating of the automation in food industries enhanced the quality of the products and reduced the production costs. The process to optimize the production of dry-fermented sausages with a reduced concentration of verotoxigenic *E. coli* (VEC) was reported. The influence of fermentation temperature, pH, and salt concentration on the survivability of VEC in *morr* and *salami* (fermented sausages) was evaluated, and results revealed that high salt and glucose content, low pH, and high fermentation temperature effectively reduced the viability of VEC. The storage at 4 °C also reduced the VEC viability in sausages. The results suggest that the optimization of processing conditions and raw material composition significantly improves the microbiological safety of dry-fermented sausages [73]. The strategies of VEC reduction in sausages was discussed by Holck et al. [74].

The post-processing treatment of salami at 43 °C for 24 h and freeze and thawing processes significantly reduced the shiga toxigenic *E. coli* (STEC) content. The reduction was strain-dependent, and the same reduction in the cell count was also observed in control strains. The reported post-processing conditions can be applied in various food industries to reduce the risk of STEC contamination in dry-fermented sausages [75]. Similarly, Heir et al. [76] also reported the influence of post-processing treatments (Heating at 43 °C for 24 h or 32 °C for 6 days; freezing at 4 °C for 30 days and thawing) effectively reduced the STEC load in representative dry-fermented sausages, *salami* and *morr*, and post-processing treatments were not affected by the sensory quality of the products [76]. The storage condition affects the AM1 binding capacity of *L. acidophilus* LA-5 in fermented milk products [77], and this is made possible due to the inactivity of the LAB strain during cold storage conditions. The post-fermented treatment of pepperoni-type, non-dry sausages (PNDS) showed a reduction in the added STEC concentration. Heating of PNDS up to 54.4 °C for 0.5 to 12.5 h significantly reduced the STEC content, the results were also associated with a final pH value of the PNDS [78].

### 4.3. Fermentation Process

The addition of plant extracts (*Nelumbo nucifera*, cloves, and *Ginkgo biloba*) reduced the fungal microflora in *meju*. The fungal isolates can produce aflatoxin in *meju*, and the fermentation process with plant extracts improved the quality and reduced the toxicity of *meju* [26].

The traditional preparation (fermentation, draining of water, washing, milling, and sieving) of *ogi* (a complementary diet made from maize) significantly (up to 90%) reduced the added mycotoxin levels. The process of soaking for 48 h accelerated the mycotoxin-elimination from the *ogi.* The results provided information to produce safe and mycotoxin-free *ogi* [79] (Table 2).

Other than the above mentioned methods, the use of plant extracts and metabolites of LAB strains such as bacteriocin are also employed in the food industries to prevent or reduce the formation of toxic substances and microbial contaminations [80,81].

## 5. Outbreaks

Though all the FF are vulnerable to microbial attack, fermented milk products are often contaminated by aflatoxins. Several countries (34 countries) determined that the maximum acceptable level of aflatoxin M1 (AFM1) is 0.05 mg/kg in milk. The limit range varies based on the type of products and country regulations [27]. Almost all the countries have their own regulatory bodies (Food and Drug Administrations, European Community Regulations, The Institute of Standards and Industrial Research of Iran, etc.) to maintain the food quality and safety. Even food-associated outbreaks are now noticed as distinct outbreaks. 

*Clostridium botulinum* was responsible for botulism in the 1930s, type E botulinum producing *C. botulinum* was reported in the Soviet Union and the U.S.A. The seafood was contaminated with type E [82]. In 1965, Jilin province, and Qinghai province in China experienced the type E botulism via contaminated fermented bean curd, and in the Qinghai-Tibet plateau region, contaminated raw meat was responsible for an outbreak and the type E *C. botulinum* was isolated. Subsequently, several Chinese provinces were affected by type E *C. botulinum* via contaminated fermented foods and/or raw meat. The details of carrier foods, type E botulism outbreaks (from 1965 to 2005), and mortality have been reported [44]. In 1975, about 64.13% of affected people died due to type E botulism. Overall, from 1965 to 1994, about 64% of these cases died because of type E botulism in China. In some areas, the mortality rate was above 80%. The strain isolated from the contaminated samples was *Clostridium butyricum*. The type E botulism outbreaks occurred mostly in the terrestrial regions of China, away from the ocean, but in the rest of world botulism mostly arises from seashore regions [44].

There was a type b botulism outbreak in Taiwan in 2006, which caused lethal effects to victims as well as dysphagia, blurred vision and even severe neurological defects happened to one victim. The poison spread via fermented foods that showed a positive type B botulism toxin. Further, review proved that the outbreaks in Taiwan from 1985 to 2006 were spread by canned and fermented foods [83]. An outbreak of foodborne botulism was reported in Surat Thani Province, Thailand in 2012. The strain isolated from the patients was found to be *C. botulinum* type B2. Previously, in 1996 and 2006, about 9 and 209 botulism cases were recorded in Thawanpha District, and Banluang District in Thailand, respectively, due to the consumption of contaminated fermented bamboo shoots [84].

Danish Statens Serum Institut, the Danish Food Safety Authority, and the Danish Food Institute investigated the frequency of Shiga toxin-producing *Escherichia coli* (STEC) isolates. The detailed research and interviews with affected people revealed that the non-O157 STEC strain STECO26 was the reason for the outbreaks in 2007 via contaminated fermented beef sausage [85]. 

In 2006, a major outbreak was documented in Yunan, China. The consumption of fermented black beans (Douchi) caused acute symptoms of nausea, vomiting, and diarrhea. The later investigation proved that the Douchi was contaminated with the *Bacillus cereus* strains. The strain characterization revealed that two strains belonged to genetic group III (ST26), and one of the strains was a strong cereulide, a major emetic toxin produced by *B. cereus*. The third strain was discovered to be enterotoxigenic *B. cereus*, which belongs to the genetic group IV. *B. cereus* strains in the contaminated Douchi can produce cereulide at 37 °C, so after consumption, toxin production may occur in the human gut, which increases the toxicity and causes adverse effects to the consumers [86].

The starter cultures used to prepare the FF products compete with the *Staphylococcus aureus* and lower the growth of *S. aureus* and staphylococcal enterotoxins (SE) production [87]. In 2014, an outbreak of staphylococcal food poisoning occurred due to the intake of Tomme (soft cheese prepared using raw cow milk) by the children and staff at a Swiss boarding school. The soft cheese contained low SE A and high SE D levels, revealing that the raw milk was contaminated with *S. aureus* [88]. 

## 6. Conclusions and Future Perspectives 

Traditionally fermented foods are commonly used in the routine diets of several people. Foodborne pathogens and food toxins are two of the leading causes of health problems. Pathogenic microbes, mycotoxins, bacterial toxins, and unwanted biomolecules (like biogenic amines) are the major spoilers of food materials, especially FF. Most raw materials used to produce FF are a source of toxic materials. For example, cereals (wheat, oat, barley) harbor several mycotoxins (deoxynivalenol, zearalenone, and aflatoxins) and fungal pathogens (*Aspergillus*, *Fusarium*, and *Penicillium* spp.). Several foodborne outbreaks have been reported. 

Recent studies demonstrated that the use of specific starter cultures (composed of LAB strains is preferable), optimized fermentation conditions, and standardized pre- or post-processing treatments effectively prevent and/or reduce the toxic materials in FF. Application of advanced automation in the food industry prevents human contact with the food material during production and post-production processing, which significantly reduces the risk of food contamination and spoilage. The improvement in the traditional fermentation processes such as post-processing (heating, freezing, and thawing) can preserve the ancient flavor of the foods and enhance food safety. Some studies suggest that simply following the traditional preparation methods (long term soaking, repeated washing) is sufficiently effective to prevent the formation of toxic materials in the food. Although the quality control measurements are improved with advanced and efficient detection methods, additional innovations are required to predict and prevent toxicity in FF. 

## Figures and Tables

**Table 1:** The reported mycotoxins and bacterial toxins in fermented foods.

S. No.	Fermented Products/Raw Materials	Toxic Compounds	Microbes	Ref.
Mycotoxins				
1	South African alcoholic beverages; Grains	Aflatoxins (200–400 µg/L), Zearalenone, Ochratoxin A	*Aspergillus* spp., *Penicillium* spp., *Mucor* spp., *Rhizopus* spp.	[10]
2	Attieke (an Ivorian fermented cassava product)	Ochratoxin (0.2 µg/kg), deoxynivalenol	-	[11]
3	Nigerian foods	Ochratoxin A (>5 µg/kg)	-	[12]
4	Wine, cider samples	Alternariol, Alternariol methyl, Ochratoxin A, penicillic acid	-	[15]
5	Nigerian foods (*iru* and *ogiri*) and raw materials	Aflatoxin	*Bacillus anthracis*, *Staphylococcus sciuri* subsp. sciuri, *Alcaligenes faecalis*, *Proteus mirabilis*	[22]
6	Southwest Nigerian foods	Fumonisin B1, Aflatoxin B1, Sterigmatocystin	-	[23]
7	*Meju* (Fermented soybean)	Aflatoxin B1, Ochratoxin A	*Agaricaceae, Mucor, Penicillium, Aspergillus, Paecilomyces.* *Aspergillus ruber*	[26]
Bacterial toxins				
8	*Soumbala* (fermented seeds of *Parkiabiglobosa*)*Bikalga* (fermented seeds of *Hibiscus sabdariffa*)	Enterotoxins	*Bacillus subtilis, B. cereus., B. licheniformis, B. pumilus*	[28]
9	*Yanyanku* and *Ikpiru* (fermented seeds of *Hibiscus sabdariffa*)	Enterotoxigenic microbes	*B. subtilis, B. cereus, B. amyloliquefaciens, B. licheniformis, B. safensis, B. altitudinis, B. aryabhattai, B. flexus, B. circulans*	[29]
10	*Yellow-Water*	Emetic toxin	*B. cereus*	[30]
11	*Gergoush*	Enterotoxigenic microbes	*Bacillus* spp.	[31]
12	*Doenjang, ssamjang, kochujang*, and *cho-kochujang*	Enterotoxins	*B. cereus sensulato*	[32]
13	*Doenjang*	Emetic toxin	*Bacillus cereus*	[33,34]
14	Fresh and fermented dairy products of Iran	Shiga toxin	Shiga toxin producing *Escherichia coli*	[35]
15	Fresh and fermented dairy products of Nigeria	Shiga toxin	Shiga toxin producing *Escherichia coli*	[36]
16	Dairy products of East and West Africa	Toxic shock syndrome toxin and enterotoxins	*Staphylococcus aureus*	[37]
17	*Ogi, nono, wara, kunu, iru*, and *kindirmo*	Enterotoxins	Coagulase-negative staphylococci strains	[38]
18	*Iru* and *ogiri*	Enterotoxigenic microbes	*B. cereus*, *Alcaligenes faecalis, Proteus mirabilis, Staphylococcus sciuri* subsp. *sciuri, B. anthracis*	[22,39]
19	*Rakfisk, faseikh, kapchunka*, and *tofu, ashbal*	Botulinum	*Clostridium botulinum*	[40]

**Table 2:** Strategies to prevent or reduce the toxic contaminants in fermented food materials.

S. No.	Fermented Foods	Toxins/Contaminants	Prevention Methods	Results	Ref.
Use of Lactic acid bacteria					
1	Fermented cerealgruels	Aflatoxin producing *Aspergillus* spp. (APA)	*Lactobacillus* spp.	*Lactobacillus* spp. inhibited the growth of APA effectively	[59]
2	Fermented maize meal (FMM)	Fumonisin B1, Zearalenone (ZEA), Aflatoxin B1 (AB1)	*Streptococcus lactis*, *Lactobacillus delbrueckii*	Reduced the B1 and ZEA content in FMM	[60,61]
3	Indigenous fermented foods of Ghana	AB1	*Saccharomyces cerevisiae*	Surface binding of AB1	[62]
4	Kefir	Aflatoxin M1 (AM1)	*Lactobacillus casei*	Surface binding of AM1	[63]
5	Fermented soybean	*Bacillus cereus* and its toxins	*Bacillus subtilis* HJ18-4	Suppressed the growth and expression of toxin-coding genes of *B. cereus*	[64]
6	Fermented soybean	*Bacillus cereus* and its toxins	*Bacillus amyloliquefaciens* RD7-7	Suppressed the growth and expression of toxin-coding genes of *B. cereus*	[65]
7	Fermented foods	ZEA	*Lactobacillus* *Plantarum* strains	Degraded the ZEA that was added in the medium	[66]
8	Fermented fish sausage	Biogenic amines (BA)	*Lactobacillus sakei* KM5474, *L. plantarum* KM1450	Reduced the amount of BA in fermented sausage	[67]
9	Fermented soybean	Tyramine	*B. amyloliquefaciens*D31-21, *B. amyloliquefaciens*O2-2, *B. subtilis* H3.	Reduced the tyramine content	[68]
10	Fermented foods	AB1	*Lactobacillus plantarum* C88	Improved the antioxidant system, facilitated the excretion of AB1, and regulated the AB1 metabolism in a rodent model	[69]
11	Korean fermented soybeans	AB1	*Aspergillus oryzae* MAO103, *A. oryzae* MAO104	Degraded the AB1 and suppressed the growth of AB1 producer	[70]
12	Sausages	*Escherichia coli*	*Staphylococcus carnosus. L. sakei*, *L. plantarum, Pediococcus pentosaceus*, *P. acidilactici*	Reduced the growth of *E. coli*	[71]
13	Fermented foods	Mycotoxins	*L. plantarum* LUHS135, *L. paracasei* LUHS244	Reduced the mycotoxins and antimicrobial activity against common foodborne pathogens	[72]
Processing conditions					
14	Dry-fermented sausages	Verotoxigenic *E. coli* (VEC)	Processing conditions	Fermentation temperature, pH, salt concentration influences the VEC survival in sausages	[73]
15	Dry-fermented sausages	Shiga toxigenic *E. coli* (STEC)	Post-processing conditions	Post-process heating, freezing and thawing, and storage conditions reduced the STEC in *salami*	[75]
16	Dry-fermented sausages	STEC	Post-processing conditions	Post-process heating, freezing and thawing, and storage conditions reduced the STEC in *salami* and *morr*	[76]
17	Fermented milk product	AM1	Storage conditions and *L. acidophilus* strain LA-5	Storage condition (4 °C for 3 weeks) affected the AM1 binding ability of *L. acidophilus*	[77]
18	Pepperoni-type sausage	Shiga toxigenic *E. coli* (STEC)	Post-processing conditions	Post-processing heating temperature, time, and final pH of the product influenced STEC content	[78]
Fermentation process					
19	*Meju*	Aflatoxin producing fungal flora	Fermentation with plant extracts	Use of plant extracts significantly reduced the fungal microflora in *meju*	[26]
20	*Ogi*	Mycotoxins	Traditional processing	Traditional processing reduced the 16 different mycotoxins in *ogi*	[79]
